# Coupled Mechanical/Dielectric Behavior of Bio-Modified PP/PBS Nanocomposites Reinforced with Organically Modified Montmorillonite

**DOI:** 10.3390/polym17223063

**Published:** 2025-11-19

**Authors:** Sirine Taktak, Nouha Ghorbel, Sébastien Rondot, Omar Jbara, Ahmed Tara

**Affiliations:** 1 LaMaCoP, Physics Department, Faculty of Sciences, University of Sfax, Sfax 3000, Tunisia; 2 MATIM, Université de Reims Champagne-Ardenne, Campus Moulin de la Housse, 51100 Reims, France

**Keywords:** biodegradable polymer, nanocomposite, correlation, dielectric properties, mechanical properties, organomodified cloisite

## Abstract

The performance of heterogeneous polymer-based materials is largely governed by the efficiency of interfacial adhesion and the strength of interactions between their constituent phases. This work mainly focuses on correlating the properties of dielectrically active interfaces, identified through broadband dielectric spectroscopy (BDS), with the mechanical behavior of heterogeneous polymer-based materials. Blends of polypropylene (PP) and biodegradable poly (butylene succinate) (PBS) were investigated across a wide composition range (100/0, 80/20, 70/30, 50/50, 20/80, and 0/100 PP/PBS). The interface between the immiscible PP and PBS phases induces a Maxwell–Wagner–Sillars (MWS) interfacial polarization in the permittivity spectrum. For the 80PP/20PBS formulation, the high activation energy of this polarization is well correlated with the material’s elevated tensile strength measured under uniaxial tension. A series of nanocomposites based on the 80PP/20PBS blend and reinforced with organically modified montmorillonite (Cloisite 20A) were thoroughly investigated. A strong correlation was established between their mechanical performance and the additional interfacial polarization arising from charge accumulation at the clay–matrix interface. The 80PP*/20PBS–3%C20 nanocomposite demonstrated superior matrix–filler adhesion, reflected by the highest activation energy of interfacial polarization and a marked increase in Young’s modulus (~22%) and zero-shear viscosity η_0_ (~44%). Complementary rheological measurements confirmed a substantial increase in viscosity and relaxation time for the 80PP/20PBS–3%C20 nanocomposites, indicating restricted chain mobility and the formation of a percolated network. Morphological analysis by SEM provided insights into the overall microstructure of the polymer blends and nanocomposites. These results demonstrate a direct correlation between interfacial structure, chain dynamics, and macroscopic performance in immiscible polymer blends and nanocomposites.

## 1. Introduction

The pursuit of sustainable materials has highlighted polymer blends as an effective strategy to develop products with tailored properties from existing polymers, enabling the combination of performance, processability, and environmental benefits. In particular, blending synthetic plastics with biodegradable polyesters provides a pathway to reduce environmental impact while maintaining or even enhancing material functionality [1,2,3].

A representative system, widely investigated by several researchers [4,5,6], is the blend of polypropylene (PP), a polyolefin, and poly (butylene succinate) (PBS), a biodegradable aliphatic polyester. PP is extensively used in applications ranging from packaging to automotive components, thanks to its excellent chemical resistance, low density, and ease of processing, yet it suffers from brittleness and low impact strength, particularly at low temperatures [7,8]. On the other hand, PBS offers high flexibility, ductility, and a large elongation at break [9,10], properties that make it an attractive candidate for improving the toughness and overall mechanical performance of polypropylene in blended systems. The combination of these two polymers therefore presents an opportunity to design sustainable materials that balance strength, flexibility, and environmental compatibility.

Moreover, polyolefins and polyesters are thermodynamically immiscible due to their distinct chemical structures and significant differences in polarity [11]. In such immiscible polymer blends, the interfaces between the two phases play a decisive role in mechanical performance, as they govern stress transfer and energy dissipation under applied loads [12,13].

To overcome the limitations associated with weak interfacial adhesion, the incorporation of nanofillers, such as layered silicates [14], carbon nanotubes [15], graphene [16], or silica nanoparticles [17], has emerged as an efficient route to compatibilize polymer blends and enhance their mechanical and thermal stability. Layered silicates, such as organomodified montmorillonite (Cloisite), have received particular attention due to their high aspect ratio, large specific surface area, and ability to promote interfacial interactions through physical or chemical bonding with the polymer matrix [14].

In addition to improving the interfacial adhesion, nanoclays can lead to a refined morphology, reduced domain size of the dispersed phase, and improved stress transfer efficiency. Furthermore, depending on the degree of exfoliation or intercalation achieved, these nanofillers can significantly influence the crystallization behavior, barrier properties, and overall mechanical response of polymer blends.

In this study, the mechanical properties through tensile tests performed at 25 °C of PP/PBS blends were evaluated for various formulations (100/0, 80/20, 70/30, 50/50, 20/80, and 0/100) by measuring tensile strength, elongation at break, and Young’s modulus. These tests highlighted the direct influence of PBS content on the mechanical performance of the blends. On this basis, the optimum formulation was selected as the host matrix for the incorporation of organomodified Cloisite (C20) at different contents (1%, 3%, 5%, and 10%), allowing for a systematic evaluation of the nanoclay’s effect on the tensile response and interfacial reinforcement of the hybrid systems.

While mechanical tests provide information on bulk properties, techniques sensitive to nanoscale phenomena are essential for directly probing the critical interfacial regions in heterogeneous polymeric matrices. In this context, broadband dielectric spectroscopy (BDS) has proven to be a powerful and highly sensitive method for investigating interfacial properties in such materials, which are often inaccessible through conventional mechanical analyses. Building upon our prior investigation, BDS was employed to study polypropylene (PP)/poly (butylene succinate) (PBS) blends with varying compositions [4]. The dielectric spectra distinctly revealed a prominent Maxwell–Wagner–Sillars (MWS) polarization process, a direct macroscopic manifestation of charge carrier accumulation at the internal PP/PBS interfaces, thereby confirming the inherent immiscibility of the two polymers. Furthermore, in the PP/PBS-based nanocomposite reinforced with organomodified Cloisite nanoclay, an additional interfacial polarization mode was identified, originating from restricted charge mobility at the polymer–clay interface [18]. A critical analysis of these interfacial polarization processes performed using the Havriliak–Negami model to determine dielectric strength and activation energy, revealed how variations in PBS content and the incorporation of high-surface-area clay affected the dielectric properties of the composites [18,19].

The central hypothesis of the current work is that these dielectrically active interfaces, previously characterized by BDS, play a decisive role in governing macroscopic mechanical performance. A second main objective of this study is to establish a strong correlation between the dielectric and mechanical properties, which provides a deeper understanding of the interfacial phenomena governing the behavior of the PP/PBS–C20 nanocomposites. By linking the characteristic parameters of interfacial polarization to the tensile response, this approach enables the identification of the mechanisms responsible for charge accumulation, interphase interactions, and their impact on the overall performance of the blends. Microscopic observations using scanning electron microscopy (SEM) were also performed to examine the morphology of the blends and nanocomposites, providing complementary insights into the phase structure and the effect of clay addition on the blend morphology. Furthermore, rheological characterizations were conducted to assess the viscoelastic behavior and flow dynamics of the materials. These analyses provided valuable information on structural organization, chain mobility, and network formation induced by clay incorporation, thereby supporting the dielectric and mechanical findings.

## 2. Materials and Methods

### 2.1. Materials

The poly (butylene succinate) (PBS) used in this work was supplied by Natureplast (Ifs, France) under the trade name PBE003. It exhibited a melt flow index (MFI) of 4–6 g/10 min (190 °C/2.16 kg) and a melting temperature of about 115 °C. The PBS showed a density of 1.26 g/cm^3^ (NF EN ISO 1183 French standard approved and published by AFNOR) and a bio-based carbon content of 51% according to ASTM D6866. The polypropylene (PP) matrix was a homopolymer provided by Repsol (reference ISPLEN PP 050Y1E). It exhibited a density of 0.902 g/cm^3^ (ISO 1183) and a melt flow rate (MFR) of 3.0 g/10 min (230 °C/2.16 kg, ISO 1133). To improve interfacial adhesion with other components, an anhydride-modified polypropylene (PP-g-MA), designated Orevac CA100 and supplied by Arkema, was employed as a compatibilizer. The MFI values were 5.8 g/10 min (230 °C/2.16 kg) for PP and 10 g/10 min (190 °C/0.325 kg) for PP-g-MA, both exhibiting a melting point close to 164 °C. The blend of PP and PP-g-MA is referred to as PP* in the following sections to differentiate it from neat PP. The nanofiller used was an organically modified montmorillonite, Cloisite 20A (C20), supplied by BYK Additives (Wesel, Germany). This clay was modified with bis(hydrogenated tallow alkyl) dimethyl ammonium to promote better compatibility with nonpolar polymer matrices. The surface treatment enhances the interlayer spacing and converts the clay layers from hydrophilic to organophilic, thereby improving their dispersion in the polymer phase. The C20 platelets are typically 1 nm thick, around 200 nm wide, and 400 nm long, with an aspect ratio of approximately 300. The basal spacing (d_001_) is 3.16 nm.

### 2.2. Experimental Processing

Before extrusion, PBS and PP-based polymers (PP and PP-g-MA) in pellet form were dry-blended with the organoclay C20, supplied as a fine powder, to ensure preliminary homogenization. The mixture was then fed into the hopper of a twin-screw extruder (Leistritz ZSE 27 MAXX, Nuremberg, Germany) operating at the laboratory scale. The extruder had a screw diameter of 28.3 mm and a length-to-diameter ratio (L/D) of 35. The processing temperatures were adjusted according to the polymer system: 130–140 °C for PBS-based formulations and 180–200 °C for PP and PP-blend compositions. The rotational screw speed was maintained at 200 rpm, with a feed rate of 3 kg/h in all experiments to ensure reproducible conditions. The content of C20 in the nanocomposites varied from 1 to 10 wt% relative to the polymer matrix. The compatibilizer PP-g-MA was incorporated at a constant 10 wt%, a proportion consistent with optimal values reported in previous studies [20]. The resulting extrudates were subsequently granulated and vacuum-dried at 80 °C for 7 h to eliminate residual moisture prior to characterization. The composition of the prepared systems are summarized in Table 1.

The twin-screw geometry consisted of a reverse-pitch melting element followed by three kneading-block sections with distinct geometries to promote distributive/dispersive mixing while limiting excessive shear; thermal degradation was prevented by using a stepwise barrel temperature profile below reported degradation onsets, controlling residence time via fixed screw speed and feed rate, and monitoring torque/melt temperature throughout processing.

### 2.3. Microstructural Analysis

A JEOL JSM-7900F field emission scanning electron microscope (FE-SEM) was employed to examine the morphological characteristics of the materials. The samples were cryo-fractured to preserve their internal structure and expose representative cross-sections. Before imaging, the fractured surfaces were coated with a thin carbon layer (~1 nm) to enhance surface conductivity and image resolution. The microscope was operated at an acceleration voltage of 15 kV and a working distance of approximately 10 mm. Micrographs acquired at a magnification of ×3000 highlighted the influence of the organoclay (C20) on the morphology of the PP*-based, PBS-based, and PP*/PBS-based matrices.

### 2.4. Mechanical Testing Procedure

The tensile behavior of the prepared materials was evaluated under uniaxial loading conditions at ambient temperature using a TEST WELL 108 Universal Testing Machine (Gennevilliers, France) equipped with a 2 kN load cell. The experiments were conducted at a crosshead speed of 10 mm/min, ensuring quasi-static deformation conditions. Test specimens were produced in accordance with the NF EN ISO 527-2 (French standard approved and published by AFNOR) Type 5A standard, employing a Babyplast 610P micro-injection molding system (Molteno, Italy). Prior to testing, all specimens were conditioned at room temperature to eliminate the influence of residual moisture or thermal history. For each formulation, a set of ten individual samples was tested to guarantee statistical reliability and reproducibility of the mechanical results. The collected data provided insight into the effect of the polymer matrix composition and nanofiller content on the tensile strength, elongation at break, and modulus of the resulting nanocomposites.

### 2.5. Dynamic Rheological Characterization

Rheological measurements of both the neat polymers, their blends, and the corresponding nanocomposites were carried out using a HAAKE MARS rotational rheometer (ThermoFisher Scientific, Waltham, MA, USA). Tests were conducted in dynamic oscillatory mode with parallel plate geometry, 25 mm in diameter and a gap of 1.5 mm, at a temperature of 180 °C. Prior to each measurement, the samples were carefully dried and molded to ensure uniform thickness and remove any residual moisture. The frequency sweep covered the range from 0.01 to 100 rad s^−1^. To ensure measurements within the linear viscoelastic region, a strain amplitude of 10% was applied to the neat matrices and blends, while a lower amplitude of 1% was used for the nanocomposites due to their higher structural sensitivity. The strain amplitude used for frequency sweeps was chosen within the linear viscoelastic region, determined from prior strain sweeps (0.01–100% at 180 °C, 10 rad·s^−1^); a criterion of ±5% variation in the storage modulus G′ relative to its plateau was applied. The linear region extended to approximately 15–20% for matrices and blends (working amplitude: 10%) and to approximately 2–3% for nanocomposites (working amplitude: 1%).

## 3. Experimental Results and Discussion

### 3.1. SEM Microstructural Observations

**Figure 1:**
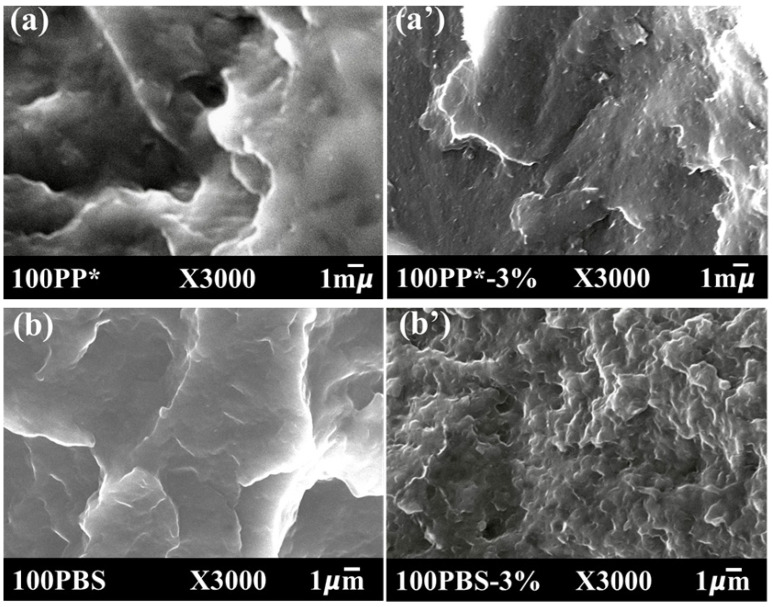

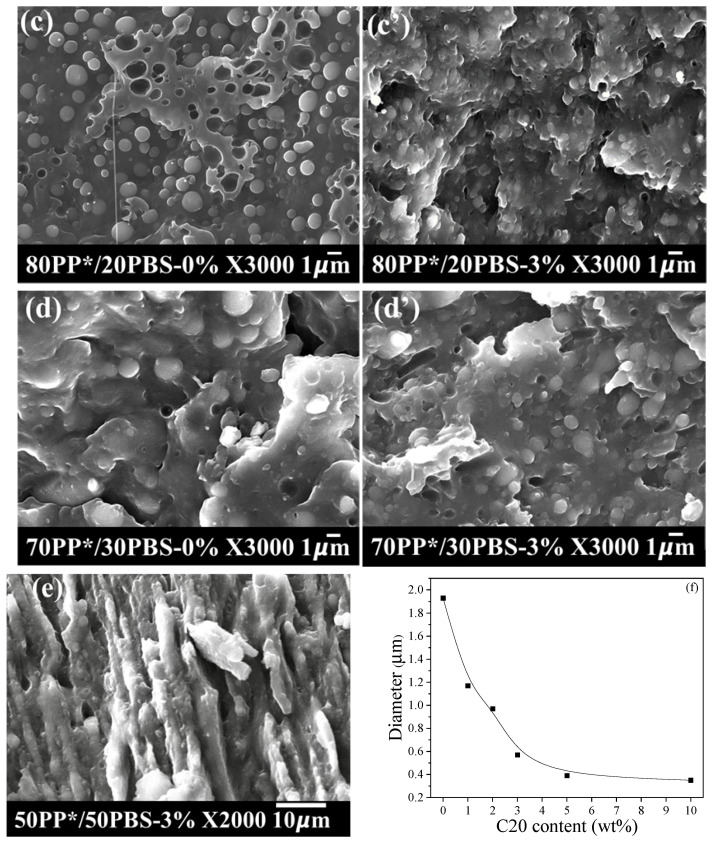
SEM micrographs of neat and 3 wt% clay-reinforced PP* and PBS, together with PP*/PBS blends at various compositions (80/20, 70/30, 50/50) either unfilled or containing 3 wt% Cloisite 20. (**a**–**d**) without Cloisite 20; (**a’**–**d’**) with 3 wt% Cloisite 20; (**e**) 50% PP / 50% PBS with 3 wt% Cloisite 20. Panel (**f**) shows the statistical analysis of PBS nodule size in the 80PP*/20PBS nanocomposite.

While scanning electron microscopy (SEM) lacks the resolution to directly probe the nanoscale dispersion state of clay platelets, it remains an essential technique for exploring the overall morphology of polymer blends and nanocomposites. Figure 1 displays SEM images of neat PP* and PBS, their nanocomposites reinforced with 3 wt% Cloisite 20, and PP*/PBS blends at compositions of 80/20, 70/30, and 50/50, including both unfilled and clay-reinforced samples.

In the polymer blend systems with ratios of 70/30 and 80/20 (Figure 1c and Figure 1d, respectively), an immiscible morphology is observed, characterized by a continuous polypropylene-rich matrix in which spherical PBS domains are uniformly dispersed. These PBS nodules, with sizes in the micrometer range, become more prominent and numerous as the PBS content increases, indicating limited interfacial compatibility between the two polymers [4,5].

The incorporation of C20 exerts a pronounced influence on the morphology of the PP*/PBS blends. As illustrated in Figure 1c’,d’, corresponding to the nanocomposites containing 3 wt% of organoclay, the overall structure remains nodular; however, the dispersed PBS domains become noticeably smaller and more uniformly distributed compared to the clay-free blends. This refinement of morphology clearly indicates that the presence of clay platelets restricts the coalescence of PBS droplets within the PP* matrix. The layered silicates are present at the polymer–polymer interface, where they serve as physical barriers and compatibilizing agents, thereby enhancing interfacial adhesion and reducing interfacial tension between the immiscible phases [21,22]. Moreover, the average diameter of the dispersed PBS nodules in the 80PP*/20PBS nanocomposite is smaller than that observed in the 70PP*/30PBS system, as shown in Figure 1c’,d’. The nodule size, measured using ImageJ software (version 1.54) for the 80PP*/20PBS nanocomposites, is presented in Figure 1f. The results clearly reveal that the diameter of the dispersed phase decreases with the incorporation of Cloisite 20 and tends to further reduce as the clay loading increases.

A more complex morphology is observed for the 50PP*/50PBS–3%C20 composition, as shown in Figure 1e. In this case, the material exhibits a combination of nodular and fibrillar structures, suggesting the onset of phase inversion and partial co-continuity between the two polymeric phases. The coexistence of nodular and fibrillar morphologies in immiscible polymer systems has been previously described [14,23,24] and is generally associated with restricted coalescence and enhanced elastic stresses during melt processing. The presence of nanoparticles may inhibit the breakup of fibrillar domains, stabilizing elongated structures and delaying their transformation into spherical droplets.

The morphological features observed in these blends and nanocomposites are expected to strongly affect their macroscopic behavior. The reduction in domain size, improved interfacial adhesion, and modified phase continuity induced by the incorporation of organoclay are likely to enhance the tensile strength and modulus of the materials, providing a more efficient stress transfer across the polymer phases.

### 3.2. Rheological Characteristics

**Figure 2:**
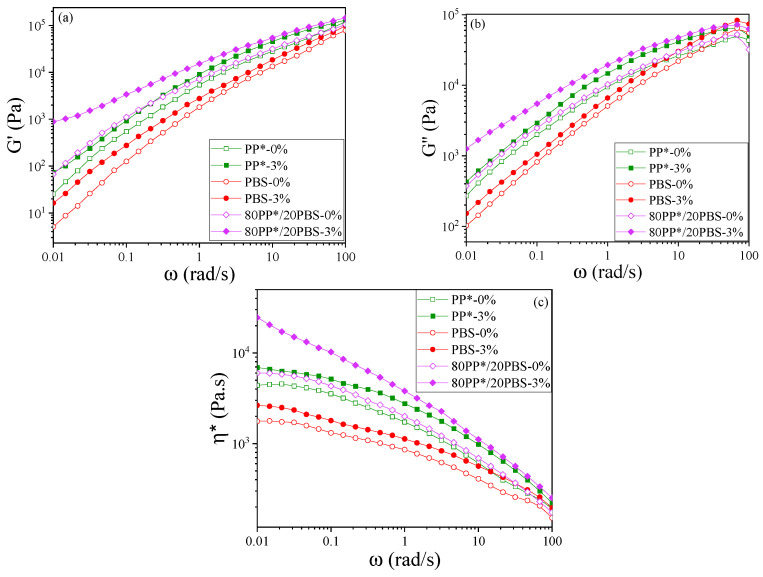
Dynamic frequency sweep results: (**a**) storage modulus G′, (**b**) loss modulus G″, and (**c**) complex viscosity η* of PP*, PBS, and 80PP*/20PBS matrices with their respective nanocomposites.

Following the morphological insights provided by SEM analysis, the viscoelastic behavior of PP*, PBS, and 80PP*/20PBS matrices, as well as their nanocomposites containing 3% C20 clay, was investigated through dynamic frequency sweeps. Figure 2a,b present the storage (G′) and loss (G″) moduli, respectively. Figure 2a illustrates the effect of blending PP* and PBS on the storage modulus (G′), showing higher G′ values for the 80PP*/20PBS blend at low frequencies compared to pure PP* and PBS. The effect of 3% clay is also evident, with G′ increasing for PP*, PBS, and the 80/20 blend at low frequencies. A similar trend is observed in Figure 2b for the loss modulus (G″).

To better interpret the influence of blending and clay incorporation on the viscoelastic response, the complex viscosity $\eta^{*}$ (defined as $\eta^{*}\;=\;\sqrt{G^{\prime 2}\;+\;G^{\prime \prime 2}\;}/\omega$) curves presented in Figure 2c were analyzed and fitted. For the pure polymers, η* as a function of angular frequency (ω) was described using the Carreau–Yasuda model [25]:

(1) $$\eta^{*}\left(\omega \right)\;=\;\;\eta_{0}\;{\left[1\;+\;{\left(\lambda \omega \right)}^{\alpha }\right]}^{\frac{n-1}{\alpha }}$$

In this model, *η*_0_ represents the zero-shear viscosity, λ denotes the characteristic relaxation time, *a* is the Yasuda parameter describing the transition region, and *n* is the power-law index reflecting the degree of shear-thinning.

For the nanocomposites, the fitting was performed using a modified Carreau–Yasuda model that incorporates a yield stress term ($\frac{\sigma_{0}}{\omega }$) [26] where *σ*_0_ present the melt yield stress. Table 2 summarize the resulting fitting parameters for the PP*, PBS, and 80PP*/20PBS matrices, and their corresponding nanocomposites containing 3% C20.

The analysis of the fitted rheological parameters provides insight into how molecular structure, phase interactions, and nanoparticle incorporation govern the flow and deformation behavior of the polymers and their blends. Pure PP* and PBS exhibit fundamentally different rheological responses, reflecting their distinct molecular architecture. The semi-crystalline nature of PP*, characterized by strong chain entanglements and ordered crystalline regions, results in a moderate zero-shear viscosity ($\eta_{0}$ = 4400 Pa·s) and a relatively long relaxation time (λ = 3.2 s). This signifies a material that resists flow and exhibits notable melt elasticity, effectively storing deformation energy. In contrast, PBS, with its more flexible aliphatic polyester backbone, displays a much lower viscosity ($\eta_{0}$ = 1750 Pa·s) and a shorter relaxation time (λ = 0.90 s). This profile is indicative of high chain mobility and a rapid dissipation of stress, consistent with its propensity for easier flow and lower melting strength.

Interestingly, the 80PP*/20PBS blend exhibits a synergistic enhancement in rheological performance that deviates from a simple rule-of-mixtures prediction. The zero-shear viscosity ($\eta_{0}$ = 9400 Pa·s) exceeds that of both constituent polymers, indicating strong interfacial interactions between the continuous PP* matrix and the finely dispersed PBS domains. These interactions act as additional constraints, effectively restricting the mobility of the polymer chains and increasing the blend’s resistance to flow. This is accompanied by a pronounced pseudoplastic behavior (*n* = 0.35), reflecting a strong shear-thinning response. The low *n* value indicates that the heterogeneous microstructure resists flow at low shear but can rearrange under higher shear, facilitating deformation while maintaining overall structural integrity.

Upon incorporation of 3wt% clay, distinct rheological changes occur depending on the polymer matrix. In both PP* and PBS, the clay primarily acts as a structural reinforcement, increasing viscosity and modifying the relaxation time, which suggests that the nanoparticles form temporary crosslinking points that restrict chain mobility and alter relaxation dynamics. These findings are in agreement with previous studies reported in the literature [27,28,29].

The most pronounced behavior is observed in the 80PP*/20PBS nanocomposite containing 3% clay, where the rheological response indicates a clear transition toward a more structured and elastic state compared to the unfilled blend. The low-shear viscosity increases significantly (+44%) and a yield stress appears ($\sigma_{0}$ = 165 Pa), indicating the formation of a three-dimensional network capable of resisting deformation before flow. The increase in relaxation time (λ = 3.9 s) confirms the restriction of segmental mobility of the polymer chains and the slowing down of relaxation processes. This structuring effect is attributed to the partial exfoliation and homogeneous dispersion of clay platelets at the PP*/PBS interface, where they act as interfacial junction points [30]. Overall, the rheological findings highlight the formation of a percolated nanostructure that strengthens the melt and modifies its viscoelastic balance toward a more elastic response. Since such molecular-level structuring is expected to have a direct impact on the solid-state behavior, the following section examines how these rheological changes are reflected in the mechanical properties of the corresponding materials.

### 3.3. Mechanical Characterization

#### 3.3.1. Mechanical Characterization of PP*/PBS Blends

**Figure 3:**
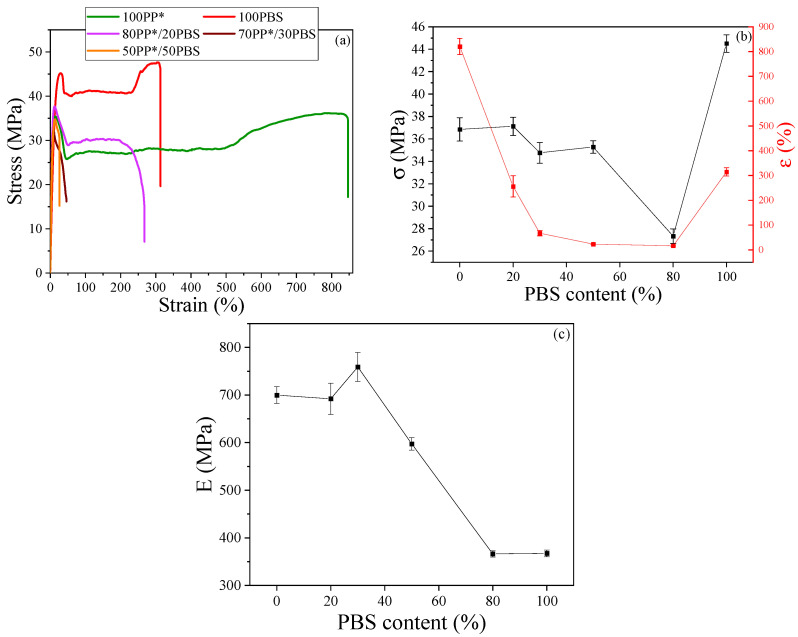
(**a**) Stress–strain curves of PP*, PBS and PP*/PBS blends, (**b**) tensile strength (left) and elongation at break (right) of PP*/PBS blends as a function of PBS content (**c**) variation in young’s modulus (E) of polymer lends as a function of PBS content.

The stress–strain curves of neat PP*, neat PBS, and their blends (80/20, 70/30, and 50/50 PP*/PBS), shown in Figure 3a, clearly illustrate the influence of blend composition on the mechanical performance of the materials. The tensile modulus (E) and ultimate strength (σ) and the elongation at the break (ε) of each sample were extracted (Figure 3b,c).

Pure PP* exhibits the characteristic ductile behavior of semicrystalline polyolefins, with a pronounced plastic deformation region and a very high elongation at break of about 820%, reflecting its excellent toughness and high chain mobility. Its relatively low tensile strength (36 MPa) and high Young’s modulus (699 MPa) indicate that while the material is initially stiff in the elastic region, it can undergo significant plastic deformation before failure, typically accompanied by necking and cold drawing. In contrast, PBS shows a higher tensile strength (44 MPa) but a much lower elongation at break (314%), demonstrating a stiffer and more brittle character. This difference arises from their distinct molecular structures: PP, composed of a flexible nonpolar hydrocarbon backbone, allows easy chain slippage under stress, whereas PBS, a polar aliphatic polyester, possesses strong intermolecular interactions and restricted chain mobility, which limit plastic flow and promote premature fracture. The enhanced deformability of PP* can also be attributed to the presence of maleic anhydride grafts (PP-g-MA), which improve interfacial adhesion and molecular compatibility, facilitating better stress transfer and contributing to a more homogeneous and ductile deformation process [31].

When PBS is incorporated into the PP* matrix, the mechanical response of the blends changes significantly. The addition of PBS leads to a noticeable increase in stiffness, attributed to the higher rigidity and crystallinity of PBS, but it concurrently causes a decrease in elongation at break and tensile strength. This trade-off results from poor interfacial adhesion between the nonpolar PP* and the polar PBS phases, which limit stress transfer across the interface. The 80PP*/20PBS composition provides an optimal balance between strength and ductility: at this concentration, the PBS domains are finely dispersed within the PP* matrix, acting as micro-reinforcing particles that restrict chain slippage and enhance stiffness without severely compromising flexibility. This morphology promotes efficient load distribution and maintains moderate elongation, leading to a synergistic mechanical response.

As the PBS fraction increases further (70/30 and 50/50 blends), the stress–strain curves become steeper, and fracture occurs at much lower strain values. This behavior reflects the progressive deterioration of interfacial compatibility and the transition from a dispersed to a co-continuous phase morphology. At these higher PBS contents, PBS domains enlarge and begin to coalesce, forming weak interphase boundaries that act as preferential crack initiation sites under tensile loading. The poor interfacial bonding restricts the ability of the PP* matrix to undergo plastic deformation and dissipate stress, resulting in a brittle fracture. Furthermore, the difference in polarity and solubility parameters between PP* and PBS promotes phase separation, as previously reported for immiscible polymer blends [10,32].

The observed mechanical trends are consistent with the morphological features revealed in previous AFM analyses, where PP* constitutes the continuous phase and PBS appears as dispersed spherical inclusions. At low PBS concentrations, these inclusions enhance stiffness through load sharing, whereas at higher contents, their agglomeration increases interfacial tension and reduces cohesive strength. Consequently, the modulus initially increases slightly at low PBS loading but decreases beyond 30–40% due to inefficient stress transfer and crack propagation at the interfaces. These findings corroborate previous studies on immiscible systems such as PLA/PP and PCL/PBS blends [33,34], which show similar composition-dependent transitions from ductile to brittle behavior.

In summary, the mechanical properties of the PP*/PBS blends are governed by the interplay between composition, morphology, and interfacial interactions. The 80PP*/20PBS formulation exhibits the most favorable combination of stiffness, tensile strength, and ductility, making it the most promising composition for applications requiring both rigidity and toughness. Increasing the PBS content beyond this threshold leads to phase separation, poor interfacial adhesion, and early failure, confirming that mechanical optimization of immiscible blends depends critically on achieving a fine and stable phase dispersion.

Furthermore, these mechanical findings are in good agreement with the rheological results, which revealed that the 80PP*/20PBS blend also exhibits an intermediate zero-shear viscosity and relaxation time, indicative of balanced chain entanglement and interfacial coupling between the two polymers. The rheological/mechanical consistency suggests that optimal viscoelastic relaxation at the molecular scale promotes efficient stress transfer and energy dissipation at the macroscopic level. Thus, both rheological and mechanical analyses converge to demonstrate that a finely dispersed morphology with moderate interfacial compatibility ensures the best balance between elasticity and toughness in immiscible PP*/PBS systems. Building on these observations, it becomes particularly relevant to explore how such interfacial organization influences other physical responses of the blends notably their dielectric behavior. The following section establishes a direct correlation between the mechanical performance and the dielectric relaxation mechanisms, providing complementary insight into the interfacial phenomena governing the PP*/PBS system.

#### 3.3.2. Correlation Between Mechanical and Dielectric Responses in PP*/PBS Blends

**Figure 4:**
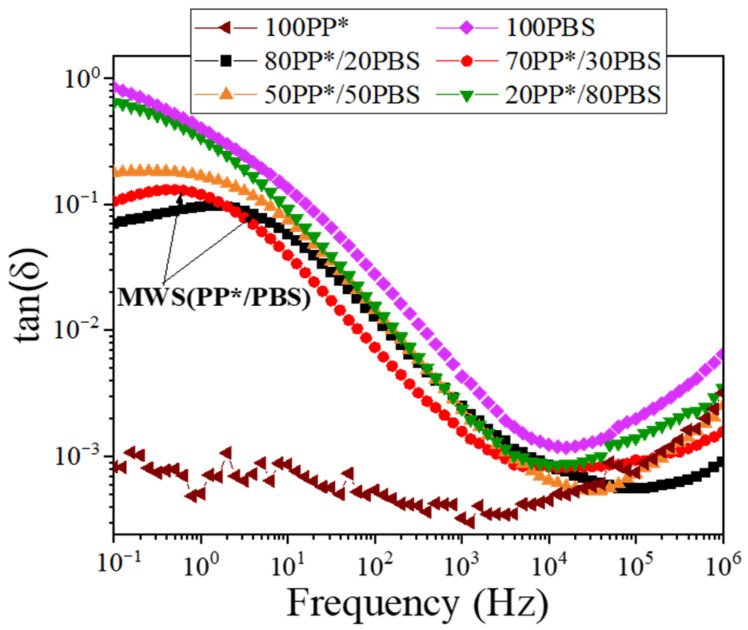
Frequency dependence of tan (δ) for neat PP*, PBS, and PP*/PBS blends.

In this section, we aim to establish a direct relationship between the rheological, mechanical and dielectric results obtained for the studied polymer blends given in our previous work [4], while the dielectric behavior of PP*/PBS blends was investigated using broadband dielectric spectroscopy (BDS). Figure 4 presents the tan δ(ω) spectra for neat PP*, PBS, and their PP*/PBS blends, illustrating the evolution of interfacial polarization as a function of blend composition. The analysis revealed a Maxwell–Wagner–Sillars (MWS) polarization arises from charge accumulation at the boundaries between the nonpolar PP* phase and the polar PBS domains providing valuable insights into the interfacial phenomena governing the PP*/PBS blends. The dielectric parameters, namely the relaxation strength (*Δ*ε) and activation energy ${(E}_{a})$, were determined using the Havriliak–Negami (HN) model and are summarized in Table 3, with the relaxation strength (Δε) measured at 60 °C and the activation energy ${(E}_{a}$) obtained from fitting the temperature-dependent data over 60 °C to 100 °C.

It is further noting that the increase in Δε from 0.52 for 80PP*/20PBS to 1.75 for 50PP*/50PBS indicates a stronger Maxwell–Wagner–Sillars (MWS) polarization, reflecting enhanced charge accumulation at poorly bonded interfaces between the nonpolar PP* and polar PBS phases. This behavior is consistent with the morphological and mechanical observations: as the PBS content increases, phase separation becomes more pronounced, interfacial adhesion weakens, and stress transfer efficiency decreases, leading to a more brittle mechanical response. In contrast, the 80PP*/20PBS blend shows the lowest Δε and the highest activation energy (0.54 eV), suggesting more restricted interfacial polarization and stronger interfacial cohesion. This improved interfacial compatibility allows both more efficient mechanical stress transfer and more homogeneous charge relaxation under an alternating electric field. Therefore, the dielectric and mechanical behaviors of the blends are strongly correlated; enhanced interfacial adhesion simultaneously promotes better load transfer and suppresses interfacial charge accumulation, confirming that both phenomena originate from the same interfacial microstructure.

#### 3.3.3. Effect of Cloisite 20 on the Mechanical Behavior of PP*, PBS, and PP*/PBS (80/20) Nanocomposites

**Figure 5:**
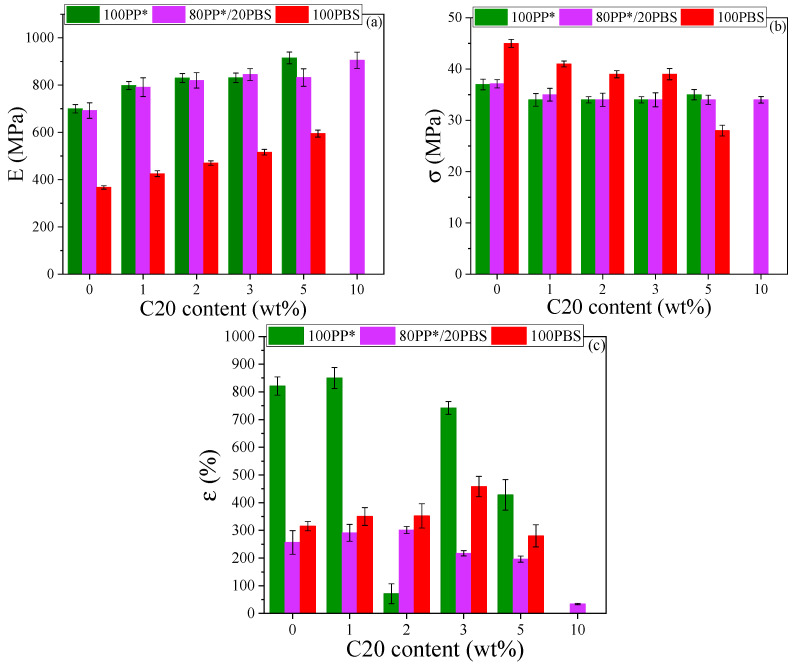
Variation in the elastic modulus (E) (**a**), tensile strength (σ) (**b**), and elongation at break (ε) (**c**) as a function of C20 content (%) for the PP*-, PBS-, and 80PP*/20PBS-based nanocomposites.

The mechanical performance of PBS/C20, PP*/C20, and 80PP*/20PBS/C20 nanocomposites were evaluated under tensile testing, and the variations in the different mechanical properties (E, σ, and ε) with nanoclay content are presented in Figure 5.

When comparing the effect of C20 on the PP* and PBS matrices, the analysis of the mechanical properties indicates that the C20 clay interacts differently with PP* and PBS, leading to distinct behaviors. The tensile modulus increases almost linearly with the addition of C20, reaching an improvement of about 30% for PP* and 62% for PBS at 5 wt% loading. This pronounced enhancement highlights the reinforcing effect of the high-modulus OMMT (C20) nanoplatelets dispersed within the polymer matrix [35]. The incorporation of these rigid nanoparticles restricts the mobility of polymer chains, resulting in a more rigid and mechanically stable structure. Such behavior reflects efficient load transfer from the polymer matrix to the dispersed clay platelets, a key indicator of good interfacial adhesion and effective stress distribution throughout the nanocomposite. For PP*, the increase in modulus is consistent, suggesting a homogenous dispersion of the nanoplatelets and the formation of an effective continuous percolation network that enhances stiffness without significant aggregation [36,37]. The relatively stable trend indicates that the nonpolar PP* matrix interacts mainly through physical confinement rather than strong chemical bonding. In contrast, PBS exhibits a much sharper rise in tensile modulus, confirming a stronger interfacial compatibility between the polymer and the organically modified clay [10]. This can be attributed to polar–polar interactions between the carbonyl groups of PBS and the organic modifier present in the C20 galleries. Such chemical affinity promotes exfoliation or intercalation of the silicate layers, leading to improved nanoparticle dispersion and enhanced interfacial adhesion [38]. Consequently, the PBS-based nanocomposites display more efficient stress transfer and a more pronounced reinforcement effect than PP*-based ones.

Concerning tensile strength and elongation at break, contrasting behaviors are observed. For PP*, the tensile strength remains relatively stable, whereas elongation at break decreases sharply at 5 % C20. This behavior is attributed to the clay acting as a nucleation site for spherulites [39], which increases their number but reduces their size. Combined with the rigidity of the fillers and the formation of agglomerates, this leads to brittle fracture. For PBS, the tensile strength decreases slightly, but ductility reaches a maximum at 3% C20. Well-dispersed nanoplatelets and effective interfacial bonding in PBS3%C20 are considered responsible for the observed increase in elongation at break. In fact, uniform nanoplatelet distribution not only stiffen the matrix but also promote plastic deformation mechanisms capable of absorbing energy. For C20 loadings greater than 3%, larger agglomerates form in the 5 wt% nanocomposites, reducing ductility. Proper dispersion of the clay is crucial to limit agglomeration and improve mechanical properties. Agglomerate formation in PBS nanocomposites at this clay loading was confirmed in our previous study [19]. Let us now focus on the selected 80PP*/20PBS system, chosen for its well-balanced combination of stiffness and ductility, and examines the effect of Cloisite 20 on its mechanical behavior. The variations in tensile strength, elongation at break, and Young’s modulus with clay content are presented in Figure 5.

The analysis of the mechanical behavior of the 80PP*/20PBS blend as a function of C20 nanoclay loading reveals that the incorporation of clay does not significantly affect the tensile strength (Figure 5b). In contrast, the elongation at break increases up to about 2% clay content, then gradually decreases before dropping sharply at 10% (Figure 5c). Meanwhile, the Young’s modulus increases steadily, reaching its maximum around 3%, confirming a steady enhancement in stiffness (Figure 5a). This mechanical behavior reflects complex interfacial mechanisms driven by the polarity differences between the components. The apolar nature of PP*, composed of saturated hydrocarbon chains, limits its interactions with the organophilic clay, whereas PBS, containing polar ester groups, exhibits a significantly higher natural affinity for the silicate surfaces of Cloisite C20 [40]. This difference in compatibility leads to a preferential selective distribution of clay nanoplatelets in the PBS phase at low loadings (1–3%) [5,18] where the clay plays a crucial role as a reactive interfacial compatibilizer by migrating toward the PP*/PBS interface. At this strategic location, the clay nanoplatelets form physical bridges that enhance adhesion between the immiscible phases: their organic modifiers interact with the aliphatic chains of PP* via London dispersion forces, while the negatively charged silicate surfaces establish dipole–dipole interactions with the carboxyl groups of PBS [41,42]. This unique configuration allows the clay to reduce interfacial tension and refine the blend morphology by decreasing the size of PBS domains (as shown by SEM), thereby creating a more stable and robust interface. As a result, mechanical load transfer is optimized, explaining the progressive increase in Young’s modulus (from 692 to 845 MPa at 3% C20), while ductility is preserved, or even slightly enhanced, due to the ability of well-dispersed nanoplatelets to align under stress and dissipate energy via micro-deformation mechanisms [43]. However, beyond the critical threshold of 3%, the progressive saturation of anchoring sites in the PBS phase and at the interface forces excess clay to form micrometer-sized aggregates in the PP* phase, where the lack of significant chemical interactions turns these aggregates into critical defects initiating cracks. At 10% loading, the formation of a percolating network of interconnected aggregates creates preferential pathways for rapid crack propagation, causing a sudden transition from ductile behavior (elongation 280–290%) to brittle behavior (34%), typical of materials overloaded with poorly dispersed nanofillers.

**Figure 6:**
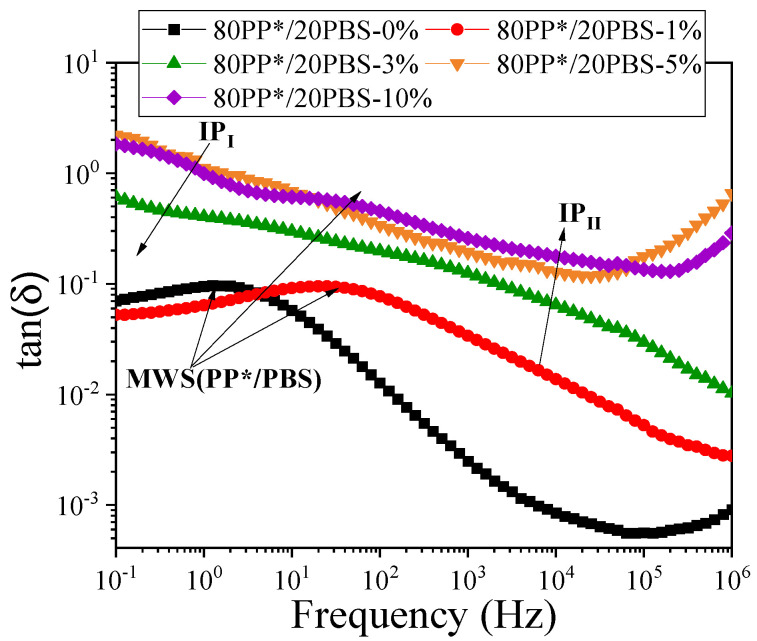
Tan δ(ω) spectra of neat 80PP*/20PBS blend and its nanocomposite containing nanoclay.

These results suggest that the optimal concentration of 2–3% C20 represents a balance point, where the benefits of nanometric reinforcement and interfacial compatibilization maximize mechanical performance without inducing the detrimental effects of aggregation. At low clay loadings, the observed increases in zero-shear viscosity and relaxation times are directly correlated with the enhancements in Young’s modulus and elongation at break, demonstrating that the nanoclay not only reinforces the polymer matrix but also constrains chain mobility in a controlled manner, promoting more efficient stress transfer. Beyond this optimal range, however, the formation of aggregates reduces the effective interfacial area, locally restricting chain mobility and causing a sharp decrease in ductility. This behavior is consistent with the rheological evidence of phase inhomogeneity, highlighting the crucial role of uniform nanoparticle dispersion in simultaneously optimizing mechanical and viscoelastic properties. These mechanical and rheological observations naturally raise questions about the corresponding interfacial electrical behavior. Dielectric spectroscopy provides complementary insight into how the nanoclay modifies charge relaxation at the PP*/PBS and PBS/clay interfaces, allowing a direct correlation between interfacial structure, chain dynamics, and the observed mechanical performance. Figure 6 illustrates the tan (δ) behavior for the 80PP*/20PBS matrix and its corresponding nanocomposite, highlighting the evolution of interfacial relaxations upon clay incorporation. Analysis of the 80PP*/20PBS-based nanocomposites revealed three distinct interfacial polarization processes. In addition to the Maxwell–Wagner–Sillars (MWS) polarization observed in the neat 80PP*/20PBS blend, originating from charge accumulation at the PP*/PBS interfaces, two additional relaxations, denoted IP_I_ and IP_II_, were identified. These are attributed to charge trapping at the matrix/clay and PBS/clay interfaces, respectively. The variation in the $E_{a}$ associated with each polarization, obtained from the temperature range of 60–100 °C, as a function of clay content is summarized in Table 4.

The parallel evolution of the activation energies $E_{a}$ (MWS) and $E_{a}$ (IP_II_) reveals a synergistic interfacial reinforcement between the different phases of the composite. The simultaneous increase in $E_{a}$ (MWS) and $E_{a}$ (IP_II_) with increasing clay content indicates a concurrent strengthening of the PP*/PBS and PBS/clay interfaces. This behavior can be explained by the preferential migration of clay nanoplatelets toward the PBS phase and the PP*/PBS interface, where they form an interconnected network that reinforces the overall structure. This interfacial synergy is reflected mechanically by the continuous increase in Young’s modulus (692→905 MPa), as the nanostructured network created by the clay progressively restricts polymer chain mobility and enhances stress transfer between the phases.

In contrast, the distinct behavior of $E_{a}$ (IP_I_), which exhibits a maximum at 3% clay content (0.48 eV) followed by a decrease, reflects a critical change in the state of dispersion. This non-monotonic evolution aligns closely with the mechanical observations: the peak at 3% coincides with the optimization of ductility (maximum elongation at 2–3% clay), whereas the subsequent decrease indicates aggregation leading to brittle behavior (elongation drop to 34% at 10% clay).

In summary, the combined mechanical, rheological, and dielectric analyses highlight the critical role of interfacial structure and nanoparticle dispersion in governing the performance of PP*/PBS nanocomposites. The synergistic reinforcement observed at the PP*/PBS and PBS/clay interfaces not only enhances Young’s modulus and ductility at optimal clay loadings (2–3%) but also restricts chain mobility, as reflected in the rheological parameters. Dielectric spectroscopy confirms that these improvements are directly linked to interfacial polarization processes, with activation energies providing a quantitative measure of interface strength and connectivity. Beyond the optimal loading, nanoparticle aggregation reduces interfacial effectiveness, leading to decreased ductility and altered dielectric responses. Overall, these findings demonstrate that precise control over interfacial interactions and nanoparticle distribution is key to achieving balanced mechanical, viscoelastic, and dielectric properties in immiscible polymer nanocomposites.

## 4. Conclusions

This study provides a comprehensive understanding of the relationship between the mechanical and dielectric responses in heterogeneous polymer-based materials, focusing on polypropylene (PP), biodegradable poly (butylene succinate) (PBS), and their corresponding nanocomposites reinforced with organo-modified montmorillonite (Cloisite 20A). The investigation highlights how the incorporation of nanoclay affects both the structural organization and interfacial interactions, establishing a direct link between microstructural evolution, charge transport mechanisms, and macroscopic mechanical performance.

Morphological observations by SEM revealed a biphasic system, where PBS formed dispersed nodules within the PP* matrix across all blend compositions. The tensile behavior demonstrated that increasing PBS content reduced the rigidity of the materials and led to earlier fracture. However, the 80PP*/20PBS blend exhibited the most balanced compromise between strength and ductility. In this composition, finely dispersed PBS nodules acted as micro-reinforcing domains, improving mechanical integrity through effective interfacial stress transfer.

Dielectric spectroscopy revealed the presence of Maxwell–Wagner–Sillars (MWS) interfacial polarization in all PP*/PBS blends, attributed to charge accumulation at the PP*/PBS interfaces. Fitting the dielectric spectra with the Havriliak–Negami model enabled the determination of dielectric strength and activation energy associated with this polarization. The 80PP*/20PBS blend exhibited low dielectric strength and high activation energy, indicating strong interfacial adhesion and reduced interfacial charge mobility. This behavior correlates well with its superior tensile performance, confirming a strong relationship between dielectric and mechanical responses.

The incorporation of Cloisite 20A significantly improved the mechanical performance of both PP* and PBS matrices. At 5 wt% clay, the tensile modulus increased by approximately 30% for PP* and 62% for PBS, highlighting the effective reinforcement induced by well-dispersed nanoplatelets that restrict chain motion and enhance load transfer. The stronger enhancement in PBS stems from favorable polar–polar interactions, ensuring better nanoparticle dispersion and more efficient stress transmission.

For the 80PP*/20PBS blend, the addition of clay led to a progressive increase in Young’s modulus, reaching its maximum at 3 wt%. At this optimal concentration, SEM observations suggested that well-dispersed clay platelets reduced the PBS nodule size and improved their uniformity within the PP* matrix. This microstructural refinement enhanced interfacial compatibility and stress transfer efficiency. The observed improvement in elongation at break at low clay contents, followed by a decrease at higher loadings, reflects the dual effect of nanoparticle-induced reinforcement and restricted chain mobility.

Dielectric analysis of the 80PP*/20PBS-based nanocomposites revealed the emergence of two additional interfacial polarizations (IP_I_ and IP_II_), in addition to the MWS polarization of the PP*/PBS interface. These were attributed, respectively, to charge accumulation at the PP*/PBS–clay and PBS–clay interfaces. The fitted dielectric parameters indicated a progressive increase in interfacial rigidity with increasing clay content, reaching a maximum for the 3 wt% nanocomposite, correlating well with the highest Young’s modulus observed for this composition.

Complementary rheological measurements confirmed this structural reinforcement. The 80PP*/20PBS–3% clay nanocomposite exhibited a 44% increase in zero-shear viscosity, a clear yield stress, and an extended relaxation time, reflecting restricted chain mobility and the formation of a percolated clay–polymer network. This interconnected structure enhances both viscoelastic strength and dielectric stability.

Overall, the 80PP*/20PBS–3% Cloisite 20A nanocomposite emerged as the most promising formulation, combining optimized mechanical strength, improved interfacial adhesion, and stable dielectric behavior. These synergistic effects make it a strong candidate for advanced applications in electrical insulation and high-performance biodegradable polymer systems.

In conclusion, this study demonstrates that the careful control of interfacial interactions and nanoparticle dispersion is key to tailoring the mechanical, rheological, and dielectric performance of immiscible polymer blends. The insights gained here provide a foundation for designing multifunctional polymer nanocomposites with optimized properties, bridging microstructural understanding and macroscopic performance. These findings open the way for future developments in high-performance, sustainable polymer systems for applications ranging from electrical insulation to biodegradable structural materials.

## Figures and Tables

**Table 1:** Matrix formulation and designation.

Name	PP (wt%)	PPgMA (wt%)	PBS (wt%)
PBS	0	0	100
PP*	90	10	0
80PP*/20PBS	70	10	20
70PP*/30PBS	60	10	30
50PP*/50PBS	40	10	50
20PP*/80PBS	10	10	80

**Table 2:** Rheological parameters from the Carreau–Yasuda model (R^2^ = 0.999) for PP*, PBS, the 80PP*/20PBS blend, and their 3% loaded nanocomposites.

	PP*		PBS		80PP*/20PBS	
	0%C20	3%C20	0%C20	3%C20	0%C20	3%C20
*σ*_0_ (Pa)	0	20	0	15	0	165
*η*_0_ (Pa·s)	4400	5800	1750	1900	9400	13,500
λ (s)	3.2	1.7	0.90	0.75	2.9	3.9
α	0.7	0.75	0.46	0.61	0.39	0.58
*n*	0.46	0.38	0.5	0.49	0.35	0.34

**Table 3:** Dielectric relaxation strength (Δε at 60 °C) and activation energy ($E_{a}$ from HN fit over 60–100 °C, 9 points) of MWS (PP*/PBS) interfacial polarization for the different PP*/PBS blends.

Name	*Δ*ε	Ea
50PP*/50PBS	1.75	0.40 ± 0.010
70PP*/30PBS	0.72	0.45 ± 0.005
80PP*/20PBS	0.52	0.54 ± 0.008

**Table 4:** Activation energies of the nanocomposites from HN fit over 60–100 °C (9 points).

Name	Ea (MWS)	Ea (IP_I_)	Ea (IP_II_)
80PP*/20PBS-0%	0.54 ± 0.008	-	-
80PP*/20PBS-1%	0.54 ± 0.007	0.35 ± 0.006	0.40 ± 0.003
80PP*/20PBS-3%	0.58 ± 0.004	0.48 ± 0.006	0.50 ± 0.003
80PP*/20PBS-5%	0.66 ± 0.001	0.42 ± 0.003	0.53 ± 0.003
80PP*/20PBS-10%	0.69 ± 0.003	0.36 ± 0.001	0.6 ± 0.006

## Data Availability

The original contributions presented in this study are included in the article. Further inquiries can be directed to the corresponding author.

## Abbreviations

The following abbreviations are used in this manuscript:

PP	Polypropylene
PP-g-MA	Polypropylene grafted with Maleic Anhydride
PBS	Poly (butylene succinate)
PLA	Poly (lactic acid)
PCL	Poly (ε-caprolactone)
MFI	Melt Flow Index
C20	Cloisite20
BDS	Broadband Dielectric Spectroscopy
FE-SEM	Field Emission Scanning Electron Microscope
G′	Storage modulus
G″	Loss modulus
*η**	Complex viscosity
*ω*	Angular frequency
λ	Relaxation time
α	Yasuda parameter
n	Power-law index
*σ*_0_	Melt yield stress
*σ*	Tensile strength
ε	Elongation at break
E	Young’s modulus
MWS	Maxwell–Wagner–Sillars
IP	Interfacial Polarization
HN	Havriliak–Negami
Ea	activation energy
*Δ*ε	Relaxation strength
